# Second and third trimester estimation of gestational age using ultrasound or maternal symphysis‐fundal height measurements: A systematic review

**DOI:** 10.1111/1471-0528.17123

**Published:** 2022-03-10

**Authors:** Alice Self, Lama Daher, Michael Schlussel, Nia Roberts, Christos Ioannou, Aris T. Papageorghiou

**Affiliations:** ^1^ Nuffield Department of Women's & Reproductive Health University of Oxford Oxford UK; ^2^ UK EQUATOR Centre, Centre for Statistics in Medicine, Nuffield Department of Orthopaedics, Rheumatology and Musculoskeletal Sciences University of Oxford Oxford UK; ^3^ Bodleian Health Care Libraries University of Oxford Oxford UK; ^4^ Oxford Maternal & Perinatal Health Institute, Green Templeton College University of Oxford Oxford UK

**Keywords:** biometry, due date, gestational age, growth, post‐term, pregnancy, pregnancy dating, preterm, screening, ltrasound dating

## Abstract

Many vulnerable women seek antenatal care late in pregnancy. How should gestational age be determined? We examine all available studies estimating GA >20 weeks. Ultrasound is much better than fundal height, and using cerebellar measurement appears to be the most accurate.

**Linked article:** This article is commented on by Philip J. Steer, pp. 1459 in this issue. To view this minicommentary visit https://doi.org/10.1111/1471‐0528.17127.

## Abstract (ro)

**Background:**

Accurate assessment of gestational age (GA) is important for managing pregnancies at an individual level and monitoring preterm birth rates at a population level.

**Objectives:**

As many women first seek antenatal care in late pregnancy, our aim was to assess the methodology of studies reporting equations for estimating GA after 20 weeks’ gestation using ultrasound or symphysis‐fundal height (SFH) measurements.

**Search strategy:**

Six electronic databases were searched for studies published from January 1970 to April 2021.

**Selection criteria:**

Studies were included if they contained a formula using SFH or ultrasound‐measured biometry to estimate GA after 20 weeks in healthy singleton pregnancies.

**Data collection and analysis:**

Two reviewers and a statistician reviewed study design, statistical methods, and reporting methods using 29 criteria. Each article was awarded an overall quality score, predefined as the percentage of the 29 criteria scored at low risk of bias. 95% prediction intervals were calculated for studies that used recommended first trimester dating to confirm true GA.

**Main results:**

The search yielded 4209 results. Ninety‐seven full‐text articles were included in the analysis. The mean quality score was 32% (range 7%–97%). Only 10 articles scored low risk in 18 or more criteria. Their formulas estimated GA using one or more ultrasound‐measured biometry parameters and SFH measurements. Twenty‐three articles used recommended first trimester dating. A single‐parameter formula using transcerebellar diameter (TCD) gave the lowest 95% prediction interval.

**Conclusions:**

There is considerable methodological heterogeneity in studies developing equations for estimating GA. Formulas using ultrasound‐based measurements more accurately estimated GA after 20 weeks than formulas using SFH measurement. While the clinical priority remains promotion of early engagement with antenatal care, we suggest unified standards for GA and growth assessment.

## 1 INTRODUCTION

Accurate assessment of gestational age (GA) is important at an individual level to manage pregnancy care appropriately and at the population level to monitor rates of GA‐dependent outcomes such as the proportion of preterm births and small‐for‐GA neonates. Although a woman’s last menstrual period (LMP) is usually used to estimate GA when ultrasound assessments are unavailable, relying on this method is problematic. For example, up to 45% of women attending antenatal care are uncertain of their menstrual dates,^1^ and LMP dating has a 95% prediction interval of ±4.65 weeks.^2^

All usual methods of GA estimation start with the fundamentally flawed assumption of equating fetal size with GA. This is practicably acceptable in the first trimester but, even then, it has been suggested that growth restriction can be observed as early as 5–10 weeks’ gestation.^3^

Nevertheless, in pregnancies conceived naturally, measurement of fetal crown‐rump length (CRL) before 14 weeks is considered the gold standard for dating pregnancies. A CRL measurement up to 84 mm is accurate to within ±5 days in 95% of cases.^4^, ^5^ When the CRL is greater than 84 mm, consensus on how to estimate GA is less clear. Although head circumference (HC) is commonly used,^6^ multi‐parameter formulas may be more accurate than a single parameter in the second and third trimesters.^4^

Dating pregnancies after 14 weeks is particularly relevant for low‐ and middle‐income countries, where many women first seek antenatal care after 20 weeks of pregnancy. In South Africa, 53% of women receive no first trimester antenatal care^7^ and up to 80% of women attend their first antenatal visit after 20 weeks in some regions.^8^ Similarly, the median age for first seeking antenatal care in Uganda is >20 weeks and only 29% receive antenatal care before their fourth month of pregnancy.^9^ This pattern of late first antenatal appointments and limited access to ultrasound^10^ hinders both optimal management of pregnant women and accurate estimates of preterm birth and small‐for‐GA neonates in regions with the highest burden.^11^

Although policy development should focus on encouraging first trimester engagement with antenatal care, statistics from high‐income countries suggest that there will always be a significant proportion of women who do not access antenatal care until later in gestation. Although early engagement with antenatal care and first trimester ultrasound screening are well‐established in England, over 35 000 (5.9%) of pregnancies ‘book’ after 20 weeks’ gestation.^12^ These women are more likely to come from minority ethnic groups and vulnerable groups, such as those with more complex psychosocial needs.^13^ In many such women, GA will often be estimated by ‘reversing’ an HC growth chart intended to describe fetal size at a given GA, which is incorrect.

At present the most methodologically robust and clinically accurate means for estimating late GA are not known. Our review aimed to close this knowledge gap by examining all studies using ultrasound or maternal symphysis‐fundal height (SFH) for estimating GA after the first trimester. We assessed these studies’ methodological quality to identify those at the lowest risk of bias and therefore most likely to develop an accurate equation for estimating GA. We also compared the accuracy of the equations developed in those studies that compared predictions with first trimester GA estimated with recommended methods.

## 2 METHODS

### 2.1 Search strategy

This systematic review of observational studies was based on study protocols previously used in our group.^14^, ^15^ It was prospectively registered in the PROSPERO international register of systematic reviews (registration number: CRD4201913776).

We conducted an electronic search of six electronic databases for the period January 1970 to 12 April 2021 to identify studies that used SFH or ultrasound‐measured biometry to estimate GA after 20 weeks’ gestation: MEDLINE (OvidSP), Embase (OvidSP), the Cochrane Database of Systematic Reviews (Cochrane Library, Wiley), Cochrane Central Register of Controlled Trials (Cochrane Library, Wiley), Science Citation Index (Web of Science Core Collection) and Conference Proceedings Citation Index (Web of Science Core Collection). We also examined the reference lists of all retrieved full‐text articles for relevant citations.

The search strategy was developed by a professional information specialist (NR) and performed with free‐text terms and medical subject headings related to GA, ultrasound, fetal development, and second and third trimesters of pregnancy (Appendix S1). Animal studies were excluded but no further limits were applied to the search. The results were imported into Endnote X9 for de‐duplication of records before screening.

### 2.2 Study selection

Two reviewers (AS and LD) screened all titles and abstracts identified to select potentially eligible studies. Consensus on any disagreements was reached by discussion with a third reviewer (AP). The two reviewers independently assessed the full texts of the selected articles to identify those that should be included. Articles were included if they contained an original formula for estimating GA in healthy singleton pregnancies calculated from fetal biometry or maternal SFH after 20 completed weeks of gestation. As our aim was to estimate GA in the late second and third trimesters; articles were excluded if formulas did not extend beyond 20 weeks. Authors were contacted for clarification if the reported formulas were not clear. Articles assessing GA in specific subgroups of fetuses, such as those with congenital abnormalities or growth aberrations, were excluded. Full‐text inclusion was limited to English, French, German and Chinese.

### 2.3 Data extraction and quality assessment

We assessed the quality of the included studies using a tool adapted from QUADAS and our previous work.^14^, ^15^ We assessed 29 quality criteria for ultrasound studies and 28 criteria for SFH studies, covering three domains: study design, statistical methods and reporting (Table S1). All study details were entered into an excel spreadsheet (Microsoft Office 365). Each criterion was scored as having high or low risk of bias by two assessors (AS and LD). Statistical methodology was also reviewed by a statistician (MS). Any discrepancies were resolved by consultation, or with another reviewer (AP or CI). The overall quality score for an article was defined as the percentage of methodological quality criteria scored as low risk of bias.

### 2.4 Assessment of the accuracy of GA prediction

We did not exclude studies from the methodological quality assessment that did not undertake first trimester ultrasound as the gold standard for estimating due date. Instead, we performed a sub‐analysis including only those studies that compared predictions with a ‘true’ GA calculated from a GA dated before 14 weeks by CRL, LMP corroborated by CRL, or IVF. This sub‐analysis compared the accuracy of the formulas that each study developed to assess GA. If a study reported several formulas, we included only the recommended formula or the formula with the lowest prediction interval.

We calculated 95% prediction intervals, in days, relative to the gold standard GA assessment using the equation of the standard deviation (SD) reported in each of the articles. Biometry measurements reported by the study, at three GA time points of clinical relevance (20, 28 and 34 weeks), were used to calculate the 95% prediction intervals using ±1.96 × SD. If an article did not report the formula that they used to calculate SD, we used their reported SDs and limits of agreement. The analysis was done using IBM SPSS Statistics for Windows, version 28 (IBM Corp.).

This review was reported following the PRISMA reporting guideline statement.^16^

## 3 RESULTS

**Figure 1:**
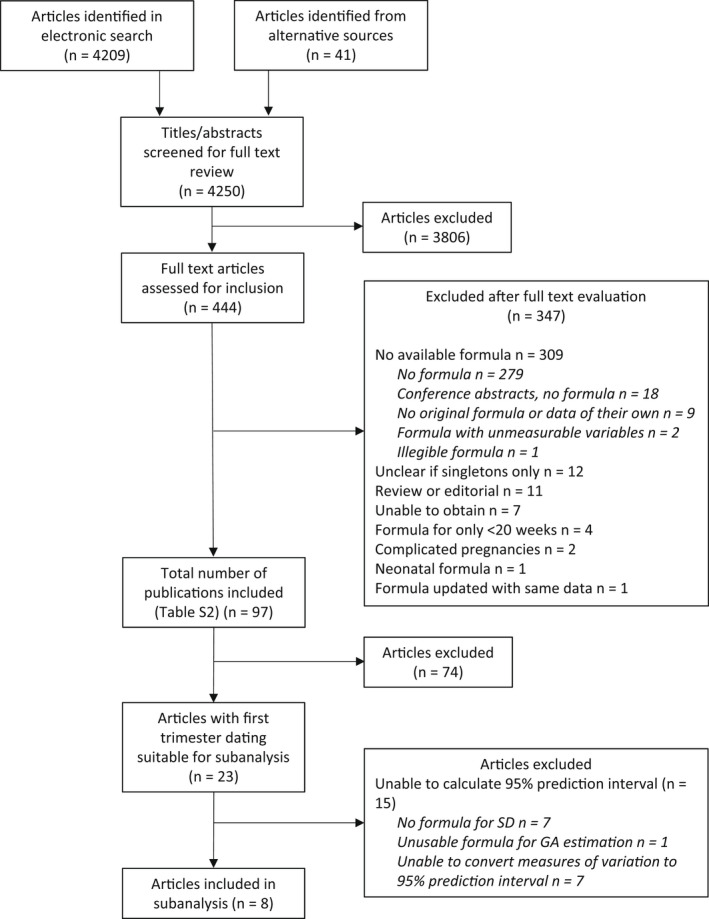
Flow diagram of study selection process

The search yielded 4209 articles, of which 403 were considered for inclusion and had a full‐text review, of which 80 were included. Another 41 articles were considered from other sources, of which 17 were included. The final analysis included 97 full‐text articles^2^, ^17^, ^18^, ^19^, ^20^, ^21^, ^22^, ^23^, ^24^, ^25^, ^26^, ^27^, ^28^, ^29^, ^30^, ^31^, ^32^, ^33^, ^34^, ^35^, ^36^, ^37^, ^38^, ^39^, ^40^, ^41^, ^42^, ^43^, ^44^, ^45^, ^46^, ^47^, ^48^, ^49^, ^50^, ^51^, ^52^, ^53^, ^54^, ^55^, ^56^, ^57^, ^58^, ^59^, ^60^, ^61^, ^62^, ^63^, ^64^, ^65^, ^66^, ^67^, ^68^, ^69^, ^70^, ^71^, ^72^, ^73^, ^74^, ^75^, ^76^, ^77^, ^78^, ^79^, ^80^, ^81^, ^82^, ^83^, ^84^, ^85^, ^86^, ^87^, ^88^, ^89^, ^90^, ^91^, ^92^, ^93^, ^94^, ^95^, ^96^, ^97^, ^98^, ^99^, ^100^, ^101^, ^102^, ^103^, ^104^, ^105^, ^106^, ^107^, ^108^, ^109^, ^110^, ^111^ published between 1974 and 2021 (Figure 1, Table S2). Most excluded studies (309 articles, including 18 conference abstracts) did not report an equation to estimate mean or median GA from given measurements or tested an existing formula rather than reporting a new formula (Figure 1).

The included studies reported data from over 100 000 women in 29 countries (median sample size 400 women, range 14–24 026, interquartile range 777).

Sixty‐nine of the 97 included articles used cross‐sectional design and analysis. Fifty‐seven of the 69 clearly stated this study design. Four of the 69 appeared to describe cross‐sectional designs but it was unclear how many times each fetus was included in the analysis. It was clear from the reporting in the other 65 articles that each fetus was only scanned or included once. Seventeen of the 97 articles used longitudinal designs and seven used mixed designs, with some women having repeat scans and others a single scan. Of these mixed and longitudinal studies, 42% (10/24) described an analysis that accounted for repeated measures. The study design for the final four articles was not clearly stated and could not be determined from the reported methods.

Sixteen of the 97 (16%) articles collected prospective data specifically for research purposes. Six reported retrospective data collection from an existing database. It was unclear whether the remaining 75 (77%) articles acquired measurements prospectively or retrospectively or whether data were collected as part of routine care or specifically for research purposes.

We identified 284 formulas for estimating GA based on 25 biometric measurements across the 97 articles (Figure S1). There were also three methods for measuring biparietal diameter (BPD). Seventy‐seven articles reported single‐parameter formulas, eight articles reported multiple‐parameter formulas, and 12 articles reported both single‐ and multiple‐parameter formulas. Only 12 (12%) reported a formula to calculate the SD of GA as the dependent variable.

The methods for defining the baseline GA (Table 1) were categorised in nine ways: LMP was the most common method (36%), followed by CRL or LMP confirmed by CRL (22%), and LMP confirmed by other ultrasound parameters but not exclusively CRL (16%).

**TABLE 1:** Methods of dating in the included studies

Types of dating	Number of studies (%)
LMP only	35 (36)
LMP confirmed by CRL or CRL only	21 (22)
LMP confirmed by US parameter (not exclusively CRL)	16 (16)
Not stated	6 (6)
LMP confirmed by T1 US	5 (5)
Mix of US parameters (CRL and other)	5 (5)
Mixed: LMP or T1 US	4 (4)
US parameter only (non CRL)	3 (3)
IVF dates	2 (2)

Abbreviations: CRL, crown‐rump length; IVF, in vitro fertilisation; LMP, last menstrual period; T1, First trimester; US, ultrasound.

**Figure 2:**
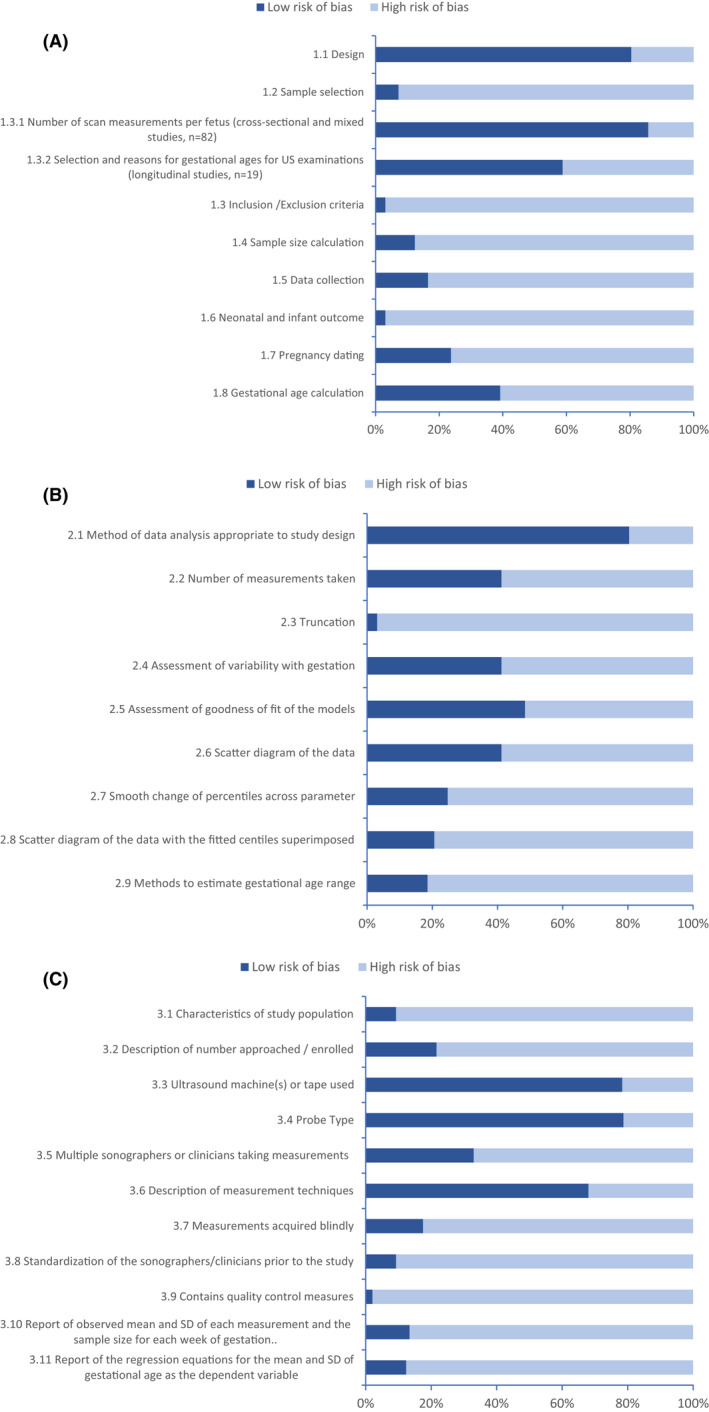
Risk of bias scores by subdomain: (A) study design, (B) statistical methods, (C) reporting methods

We could assess the risk of bias in all 97 articles. The results for each domain and criterion are presented in Figures 2A‐C. Table S3 lists each study’s detailed scores for each quality criterion. The mean quality score was 32% (range 7–97%), with 12 articles scoring more than 50% and 38 less than 25%.

Quality criteria most at risk of bias were neonatal outcomes, truncation of data, inclusion and exclusion criteria (each only described in three articles), and presence of systematic ultrasound quality control measures (fully described in two articles). Although 32 articles reported more than one sonographer or clinician taking measurements, only nine (9%) described any standardisation exercises and only two described a full set of quality control measures. Figure S2 shows the proportion of articles that reported using each possible quality control measure.

Many articles did not fully describe maternal characteristics and criteria for inclusion and exclusion. Only three articles described a complete set of recommended exclusion criteria.^14^, ^15^ Forty‐three articles reported exclusions that risked introducing bias, such as removing cases based on birthweight or outside the 10–90th centiles of measurements. Six articles clearly reported appropriate methods for excluding outliers, such as those more than 5 SD from the mean.

We identified 10 articles at low risk of bias for 18 or more criteria (≥62%). Table 2 lists their formula for calculating GA, which used one or more of abdominal circumference, BPD, HC, femur length, TCD and SFH. Of these, only the Altman and Johnsen studies^22^, ^59^, ^60^ did not use CRL dating to confirm the GA.

**TABLE 2:** Formulas and quality scores of top 10 scoring articles from lowest to highest risk of bias

Author	Formula	Quality score (%)
Papageorghiou et al. 2016^80^	exp[(0.03243 × (log_e_(HC))^2^ + (0.001644 × FL × log_e_HC) + 3.813] exp[(0.05970 × (loge(HC))2 + (0.000000006409 × HC3) + 3.3258]	97
Papageorghiou et al. 2016^81^	6.585838‐(2.7072585 × SFH^0.5^) + (1.295291 × SFH)	89
Rodriguez‐Sibaja et al. 2021^87^	3.957113 + 8.154074 × (TCD/10) – 0.076187 × (TCD/10)3	79
Skupski et al. 2017^95^	10.6 – (0.168 × BPD) + (0.045 × HC) + (0.03 × AC) + (0.058 × FL) + (0.002 × BPD2) + (0.002 × FL2) + (0.0005 × BPD × AC) – (0.005 × BPD × FL) – (0.0002 × HC × AC) + (0.0008 × HC × FL) + (0.0005 × AC × FL)	66
Altman et al. 1997^21^	exp[(0.044653 × BPDoo) – (0.0060089 × BPDoo) × logBPDoo + 1.961]	66
	exp[(0.045570 × BPDoi) – (0.0061838 × BPDoi) × logBPDoi + 1.985]	
	exp[(0.010451 × HC) – (0.000029919 × HC^2^) + (0.43156 × 10^−7^HC^3^) + 1.854]	
	exp[0.79107logTCD + 0.6439]	
	exp[(0.034375 × FL) – (0.0037254 × FL) × logFL + 2.306]	
Briceno et al. 2013^23^	24.83392 + (1.886759 × BPD) + (0.1168198 × BPD^2^) + (0.0025451 × BPD^3^)	66
	24.53281 + (9.017666 × HC) – (1.579865 × HC^2^) + (0.5223578 × HC^3^)	
	25.31365 + (5.06337 × AC) + 1922266 × AC^2^) – (0.2513339 × AC^3^)	
	exp(3.21422 + (0.2158713 × FL)‐(0.0039493 × FL^2^)‐(0.0001861 × FL^3^)	
Johnsen et al. 2005^59^	8.6245 + (1.3950 × FL^0.5^) + (0.00395 × FL^2^)	66
AMANHI 2020^68^	exp[0.3825021(lnTCD) + 0.3321277(lnFL) + 2.63416]	66
	exp[0.4390124(lnTCD) + 0.2968778(lnBPDoi) + 2.490502]	
	exp[0.4569856(lnTCD) + 0.2244807(lnAC) + 2.454795]	
	exp[0.3107083(lnTCD) + 0.2450894(lnFL) + 0.1397663(lnBPD) + 0.0626322(lnAC) + 2.297582]	
Johnsen et al. 2004^58^	exp[2.507 – (1.333 × BPD^‐0.5^) + (0.0139 × BPD)]	62
	exp[1.544 + (0.886 × HC^−1^) + (0.1103 × HC^0.5^)]	
Leung et al. 2008^64^	7.996225 + (2.277074 × BPDoi) + (0.025200 × BPDoi^2^) + (0.008007 × BPDoi^3^)	62
	7.717964 + (2.339119 × BPDoo) + (0.010324 × BPDoo^2^) + (0.008511 × BPDoo^3^)	
	6.395595 + (0.966476 × HC) – (0.019933 × HC^2^) + (0.00059 × HC^3^)	
	11.52821 + (1.591733 × FL) + (0.435641 × FL^2^) – (0.017006 × FL^3^)	

To assess GA estimation prediction intervals, we undertook a sub‐analysis of the studies that compared their formulas’ predictions to recommended first trimester GA estimates (*n* = 23). These articles had quality scores ranging from 17% to 97%. Seven articles did not report any measure of variation. An equation for the SD could only be obtained for five papers, one of which was excluded from the sub‐analysis because we were unable to recreate the GA estimation from the formulas given. Another seven articles reported measures of variation that could not be converted to a prediction interval with the data provided. Table 3 shows the results of this analysis. The half‐width 95% prediction interval was 8–21 days at 20 weeks, 11–25 days at 28 weeks, and 12–28 days at 34 weeks.

**TABLE 3:** Sub‐analysis: Table of half‐width 95% prediction intervals for equations given in articles which used gold‐standard first trimester dating

Author	Year	Parameter for GA measurement	Quality score % low risk of bias	95% PI at 20 weeks ± days	95% PI at 28 weeks ± days	95% PI at 34 weeks ± days
Single‐parameter formulae						
Biparietal diameter						
Leung et al.^64^	2008	BPDoi	62%	13	19	22
Leung et al.^64^	2008	BPDoo	62%	12	19	23
Head circumference						
Papageorghiou et al.^80^	2016	HC	97%	9	16	23
Leung et al.^64^	2008	HC	62%	10	17	21
Femur length						
Leung et al.^64^	2008	FL	62%	10	14	17
Transcerebellar diameter						
Rodriguez‐Sibaja et al.^87^	2020	TCD	79%	9	12	13
Other						
Ozat et al.^79^	2011	SaL	34%	*21* ^a^	*25* ^a^	*22* ^a^
Symphysis fundal height						
Papageorghiou et al.^81^	2016	SFH	89%	21	25	28
Multiple‐parameter formulae						
AMANHI^68^	2020	TCD and FL	66%	Not given	11^b^	15^b^
Skupski et al.^95^	2017	BPDoi, HC, AC and FL	66%	8	12	17
Papageorghiou et al.^80^	2016	HC and FL	97%	9	13	16
Sun et al.^96^	2020	BPDoi, HC, AC and FL	55%	12	17	12

*Note*: 95% prediction interval = ±SD × 1.96.

^a^ ±2SD in weeks given and midpoint between the two multiplied by 7 to give estimate of prediction interval in days.

^b^ SD not given but approximation in days reported as the midpoint between the unsigned 95% limits of agreement.

## 4 DISCUSSION

### 4.1 Main findings

In this review, we addressed the two key attributes that must be considered when identifying the best equations for estimating GA: the methodological rigour of the study developing the equation and the accuracy of the developed equation. We followed the approach of two previous reviews^14^, ^15^ to assess the methodological quality of 97 studies reporting equations for estimating GA beyond 20 weeks of gestation. We assessed the self‐reported accuracy of equations developed by studies that used optimal methods to calculate the baseline ‘ground truth’ GA using CRL or IVF dates.

At 11–14 weeks’ gestation, CRL measurements have a half‐width 95% prediction interval of around 5 days^4^, ^5^ the true GA will be within ±5 days of the estimated GA 95% of the time. At all sampled GA time points, ultrasound formulas more accurately estimated GA than SFH formulas. At 20 weeks’ gestation, the multiple‐parameter formulas from Papageorghiou et al.^81^ and Skupski et al.^96^ gave half‐width 95% prediction intervals of 8–9 days, which increased with increasing GA (Table 3). A single‐parameter formula using TCD had the lowest 95% prediction intervals.^88^

### 4.2 Interpretation

Our review highlights the considerable methodological heterogeneity of studies proposing equations for assessing GA after 20 weeks’ gestation. Only a few studies were comprehensively and rigorously reported. Our group has previously described the importance of appropriately selected populations for growth and dating studies.^14^, ^15^

Researchers should carefully consider their inclusion and exclusion criteria and selection of study participants to ensure that women and their fetuses are at low risk for growth aberrations. Once such selection is made at baseline, further exclusions should only be made for severe conditions, such as maternal or fetal death or subsequent diagnosis of a major fetal anomaly. Only three studies^81^, ^82^, ^88^ used a comprehensive list of factors known to affect fetal growth in their maternal inclusion and exclusion criteria.

Many of the included studies excluded outer percentiles of ultrasound measurements; however, it is inappropriate to exclude fetuses or neonates on size parameters such as being below the 5th or 10th centiles, above the 90th or 95th centiles, or weighing less than a specified weight at birth. Such exclusions artificially reduce the 95% prediction interval and overestimate precision, as natural variation is not fully represented.

Only two articles rigorously used quality control measures to reduce bias.^81^, ^96^ Having all scans performed by a single operator mitigates against inter‐operator variability but does not represent the clinical situation of numerous operators performing ultrasound scans. Standardised scanning procedures improve the consistency of data measurements^112^ and should be accompanied by intra‐ and inter‐operator variability assessments of the collected data. Clear protocols for saving, reviewing and scoring scans are also required.

When a woman presents after 14 weeks’ gestation, the fetal HC is commonly used to estimate GA. A widely used method involves manually or computationally plotting the HC measurement along the 50th centile of an HC growth chart and identifying the corresponding GA for that HC measurement. Several authors^113^, ^114^ have explained why it is inappropriate to estimate GA from growth charts, rather than using a formula specifically designed to estimate GA from HC. Under half of the articles reviewed included scatter plots with GA as the dependent variable, implying that this concept is not well understood.

Truncation involves excluding values outside a given window from regression analysis in post‐hoc data refinement. If biometry measurements are taken within a fixed GA range but are not further restricted before inclusion in the regression analysis, the average GA may be overestimated at the lowest extreme measurements and underestimated at the upper extremes.^22^ However, only three articles reported using truncation.

Of the 23 articles with appropriate first trimester dating, only eight were eligible for inclusion in our sub‐analysis based on correct modelling of the SD across GA (Figure 1). Most of the included articles did not adequately report the precision of their GA equations. The extent of heterogeneity in reporting precision made it difficult to compare precision across studies. Articles reported SD, standard errors (SE), confidence intervals, prediction intervals and limits of agreement. Some articles erroneously assumed that SD or SE were constant throughout gestation, even though variability in fetal growth parameters increases with gestation and simple methods are available for modelling data variability and goodness of fit.^115^

### 4.3 Strengths and limitations

Although it is widely accepted that the gold standard for dating a pregnancy is in the first trimester by a CRL measurement, LMP confirmed by a CRL measurement or IVF‐assigned dates, most of the analysed studies failed to include this requirement in their study design. Although some of the stronger arguments to stop dating pregnancies using the LMP were made in the 1990s,^116^, ^117^ it was often years before national recommendations introduced a first‐trimester scan to provide optimal dating.

We had originally planned to include only articles with optimal dating to avoid a circular argument whereby the same biometry measurements are used to date a pregnancy and estimate the GA. However, we would then have excluded most studies before the mid‐1990s and from low‐ and middle‐income countries, where first trimester scanning is less common. This change increased the number of articles and formulas included. We did not change how we assessed methodological quality or precision, and we performed a sub‐analysis of studies that only used appropriately dated pregnancies.

Although we did not place any language restrictions on the search strategy, we were only able fully to translate articles written in Chinese, French, German, Italian and Spanish. Six studies were therefore excluded. We do not anticipate this exclusion to have significantly affected our findings because this is a review of methodology and not a meta‐analysis of a treatment effect.

Sonographers should be blinded to their measurements and GA to remove observer bias;^118^ however, most studies did not report using such blinding. Ultrasound systems routinely display a measurement and estimated GA from the biometric plane of interest, which could introduce bias when constructing fetal growth charts or methods for assessing GA. Blinding the sonographer is conceptually similar to blinding the operator to the maternal SFH measurement during pregnancy assessment.

As some articles provided very limited method sections, many categories were scored at high risk of bias simply because key information was not given. We believe, however, that in most cases low‐quality reporting correlates with low‐quality methodology.

Two of the articles reporting equations with the lowest half‐width 95% prediction intervals^81^, ^88^ came from our group, and so their quality scores benefit from a greater awareness of the reporting criteria that our group has previously published.^14^, ^15^ Although this may bias the quality score results, it does not impact on the self‐reported accuracy of GA estimation.

This review has a number of strengths. The approach used to assess methodological quality has been previously tested and used.^14^, ^15^ The risk assessment criteria enabled an objective, quantitative assessment that allows studies to be compared, can easily be replicated by other groups, and can serve as a guide for designing future fetal ultrasound studies.

We did not limit this review by date of publication, as it was possible that an old formula could provide the most precise estimate of GA. However, statistical methodology in the field has advanced over time and older studies may be considered less methodologically rigorous by today’s standards. The rigour of the statistical methodology used was assessed during methodological quality assessment and is therefore reflected in the risk of bias score. Appraisal of statistical analyses was supervised by an experienced statistician.

### 4.4 Practical and clinical implications

We were able to identify the highest scoring studies with the lowest risk of bias. We analysed those that used the gold‐standard first trimester dating as their reference for estimating GA and calculated 95% prediction intervals to identify the most precise formulas. This work can inform clinical practice, and focus future prospective testing of formulas for estimating GA using an external dataset to best assess precision.

All GA estimates were more uncertain when based on SFH measurements than on ultrasound measurements. It is reasonable to assume that the best formula for estimating GA will come from a study with good methodology and the lowest self‐reported prediction interval. In our view, the combination of the most robust methods and lowest prediction intervals are the TCD formula by Rodriguez‐Sibaja^87^ and the multiple‐parameter formulas by Papageorghiou et al.,^81^ Skupski et al..^96^ and the AMANHI (WHO Alliance for Maternal and Newborn Health Improvement) Late Pregnancy Dating Study Group^69^ if a TCD measurement is not available.

Our findings of greater inaccuracy using SFH than ultrasound are of particular significance to low‐ and middle‐income countries, where the proportion of women seeking antenatal care late in pregnancy, and the burden of small‐for‐gestational age and preterm birth are highest. Many articles in our analysis included ultrasound in pregnant women from under‐served regions, and we therefore believe that the findings are generalisable worldwide. In settings where ultrasound resources are limited, it is even more important that early engagement with antenatal care is promoted, to optimise benefits from recommended ultrasound^119^ and minimise inaccuracies of late ultrasound‐based GA assessment. It can be argued that it is fundamentally erroneous to equate GA to ultrasonographic estimation of fetal size. However, when GA is unknown, there are currently no alternative methods of GA estimation ready for widespread clinical use. The vast number of formulas found in the international literature suggest the field has been exhaustively explored and that ultrasound‐measurement‐based methods have reached a plateau of possible accuracy. We propose that other methods should be explored, such as those based on machine‐learning methods^120^, ^121^ or other biomarkers, either alone or in combination with ultrasound. Nevertheless, until there are better alternatives available for use in resource poor settings, there remains a significant benefit to knowing the most accurate equations with which to estimate GA from easily measurable fetal biometric parameters.

## 5 CONCLUSION

While the clinical priority should remain promoting early engagement with antenatal care including first trimester ultrasound dating, a proportion of pregnant women will always access antenatal care later in pregnancy. This systematic review has highlighted considerable methodological heterogeneity among studies creating formulas to estimate fetal GA in late pregnancy. We identified the formulas most likely to accurately estimate GA after 20 weeks^69^, ^81^, ^88^, ^96^ using ultrasound‐derived biometry of the fetal cerebellum or multiple parameters. We also show that they are superior to dating by SFH measurement. Unified standards for GA and subsequent growth assessment should be used clinically.

## CONFLICT OF INTERESTS

Completed disclosure of interest forms are available to view online as supporting information.

## AUTHOR CONTRIBUTIONS

AS: Conceptualisation, Methodology, Data extraction and analysis, Writing – original draft, review and editing; LD: Data extraction and analysis, Writing – review and editing, MS: Methodology, Data extraction, Writing – review and editing; NR: Literature review, Writing – methods and review, CI: Conceptualisation, Methodology, Consensus in data extraction, Writing – review and editing, Supervision; AP: Conceptualisation, Methodology, Consensus in data extraction, Writing – review and editing, Supervision.

## ETHICS APPROVAL

There was no patient or public involvement in the development of this review.

## Supporting information


**Appendix** S1

**Appendix** S2

**Appendix** S3

Data S1

Data S2

Data S3

Data S4

Data S5

Data S6

## Data Availability

Data sharing is not applicable to this article as no new data were created or analyzed in this study.
