# Supplementary material for: Second and third trimester estimation of gestational age using ultrasound or maternal symphysis‐fundal height measurements: A systematic review
Source: BJOG. 2022 Mar 10;129(9):1447–58. doi: 10.1111/1471-0528.17123 (PMC9545821; doi:10.1111/1471-0528.17123)
Supplement: Supplementary file 1 — Appendix S1 [file BJO-129-1447-s009.docx]

Appendix S1: Search strategies – conducted 12/04/2021

Epub Ahead of Print, In-Process & Other Non-Indexed Citations, Ovid MEDLINE(R) Daily and Ovid MEDLINE(R) 1946 to Present

| [# ▲](https://ezproxy-prd.bodleian.ox.ac.uk:2483/ovid-a/ovidweb.cgi?&S=FLGBFPELDHEBLIFBJPPJDHBHLGICAA00&Sort+Sets=descending) | **Searches** | **Results** |
| --- | --- | --- |
| 1 | Ultrasonography, Prenatal/ | 32504 |
| 2 | (ultrasound or ultrasonogra* or ultra-sound or ultra-sonogra* or sonogra* or echograph* or echogram?).mp. or di.fs. | 3008439 |
| 3 | (fetal or foetal or fetus* or foetus or prenatal* or pre-natal*).mp. | 543980 |
| 4 | 2 and 3 | 113090 |
| 5 | 1 or 4 | 113090 |
| 6 | "embryonic and fetal development"/ or fetal development/ or gestational age/ | 108520 |
| 7 | gestational age.ti,ab. | 71641 |
| 8 | ((fetal or foetal or fetus* or foetus) adj2 (growth or development or size)).ti,ab. | 33079 |
| 9 | ((fetal or foetal or fetus* or foetus or menstrua*) adj2 (age or date or dating)).ti,ab. | 5009 |
| 10 | sga.ti,ab. | 8743 |
| 11 | 6 or 7 or 8 or 9 or 10 | 176123 |
| 12 | exp Cerebellum/ or cerebral ventricles/ | 75327 |
| 13 | (brain? or cerebell* or transcerebell* or cerebral).ti. | 543729 |
| 14 | (cerebellar or transcerebellar or cerebellum or cerebral cortex).ti,ab. | 141102 |
| 15 | ((brain or cereb*) and ((anterior or posterior) adj2 ventric*)).ti,ab. | 373 |
| 16 | ((cerebel* or transcerebel*) and diameter*).ti,ab. | 1414 |
| 17 | tcd.ti,ab. | 4235 |
| 18 | Cephalometry/ | 27182 |
| 19 | Head/gd [Growth & Development] | 741 |
| 20 | (head adj2 (circumference or biometr*)).ti,ab. | 7272 |
| 21 | ((biparietal or bi-parietal) adj2 (diameter* or measure* or biometr*)).ti,ab. | 1912 |
| 22 | (bpd adj2 measure*).ti,ab. | 304 |
| 23 | ((occipitofrontal or occipito-frontal) adj2 (diameter* or measure* or biometr*)).ti,ab. | 125 |
| 24 | (((biparietal or bi-parietal) adj5 (occipitofrontal or occipito-frontal)) and (diameter* or ratio*)).ti,ab. | 88 |
| 25 | ((fundal or symph*) adj2 height).ti,ab. | 384 |
| 26 | Abdomen/gd [Growth & Development] | 102 |
| 27 | ((abdomen or abdominal) adj2 (circumference or measure* or biometr*)).ti,ab. | 4702 |
| 28 | (ac adj2 measure*).ti,ab. | 966 |
| 29 | Femur/gd [Growth & Development] | 1286 |
| 30 | ((femur* or femoral) adj2 (length* or measure* or biometr*)).ti,ab. | 4946 |
| 31 | (fl adj2 measure*).ti,ab. | 192 |
| 32 | ((fetal or foetal or fetus* or foetus) adj2 biometr*).ti,ab. | 933 |
| 33 | 12 or 13 or 14 or 15 or 16 or 17 or 18 or 19 or 20 or 21 or 22 or 23 or 24 or 25 or 26 or 27 or 28 or 29 or 30 or 31 or 32 | 683110 |
| 34 | 5 and 11 and 33 | 4157 |
| 35 | Pregnancy Trimester, Second/ | 15235 |
| 36 | ((second or 2nd or middle) adj3 trimester?).ti,ab. | 20501 |
| 37 | Pregnancy Trimester, Third/ | 14964 |
| 38 | ((third or 3rd or late or last or final) adj3 trimester?).ti,ab. | 23220 |
| 39 | ((late or 2nd half or second half) adj2 pregnanc*).ti,ab. | 11429 |
| 40 | ((14* or 15* or 16* or 17* or 18* or 19* or 20* or 21* or 22* or 23* or 24* or 25* or 26* or 27* or 28* or 29* or 30* or 31* or 32* or 33* or 34* or 35* or 36* or 37* or 38* or 39* or 40* or 41* or 42*) adj2 week?).ti,ab. | 267042 |
| 41 | ((fourteen* or fifteen* or sixteen* or seventeen* or eighteen* or nineteen* or twenty or twentieth or thirty or thirtieth or forty or fortieth) adj3 week?).ti,ab. | 9882 |
| 42 | 35 or 36 or 37 or 38 or 39 or 40 or 41 | 316874 |
| 43 | 34 and 42 | 2642 |
| 44 | 11 and 33 | 13298 |
| 45 | limit 44 to "reviews (maximizes specificity)" | 161 |
| 46 | 43 or 45 | 2778 |
| 47 | exp animals/ not humans.sh. | 4811048 |
| 48 | 46 not 47 | 2762 |

Embase (OvidSP)[1974-present]

| [# ▲](https://ezproxy-prd.bodleian.ox.ac.uk:2483/ovid-a/ovidweb.cgi?&S=FLGBFPELDHEBLIFBJPPJDHBHLGICAA00&Sort+Sets=descending) | **Searches** | **Results** |
| --- | --- | --- |
| 1 | fetus echography/ | 24990 |
| 2 | (ultrasound or ultrasonogra* or ultra-sound or ultra-sonogra* or sonogra* or echograph* or echogram?).mp. | 852311 |
| 3 | (fetal or foetal or fetus* or foetus or prenatal* or pre-natal*).mp. | 661860 |
| 4 | 2 and 3 | 81619 |
| 5 | 1 or 4 | 81619 |
| 6 | fetus growth/ or gestational age/ | 156063 |
| 7 | gestational age.ti,ab. | 103484 |
| 8 | ((fetal or foetal or fetus* or foetus) adj2 (growth or development or size)).ti,ab. | 44578 |
| 9 | ((fetal or foetal or fetus* or foetus or menstrua*) adj2 (age or date or dating)).ti,ab. | 6715 |
| 10 | sga.ti,ab. | 14114 |
| 11 | 6 or 7 or 8 or 9 or 10 | 220263 |
| 12 | exp Cerebellum/ and diameter*.ti,ab. | 1113 |
| 13 | ((cerebel* or transcerebel*) and diameter*).ti,ab. | 1999 |
| 14 | tcd.ti,ab. | 7908 |
| 15 | Cephalometry/ | 21153 |
| 16 | head circumference/ | 13766 |
| 17 | (head adj2 (circumference or biometr*)).ti,ab. | 10415 |
| 18 | Biparietal distance/ | 1740 |
| 19 | ((biparietal or bi-parietal) adj2 (diameter* or measure* or biometr*)).ti,ab. | 2336 |
| 20 | (bpd adj2 measure*).ti,ab. | 348 |
| 21 | ((occipitofrontal or occipito-frontal) adj2 (diameter* or measure* or biometr*)).ti,ab. | 151 |
| 22 | (((biparietal or bi-parietal) adj5 (occipitofrontal or occipito-frontal)) and (diameter* or ratio*)).ti,ab. | 96 |
| 23 | ((fundal or symph*) adj2 height).ti,ab. | 546 |
| 24 | abdominal circumference/ | 3168 |
| 25 | ((abdomen or abdominal) adj2 (circumference or measure* or biometr*)).ti,ab. | 7539 |
| 26 | (ac adj2 measure*).ti,ab. | 992 |
| 27 | leg length/ | 2413 |
| 28 | ((femur* or femoral) adj2 (length* or measure* or biometr*)).ti,ab. | 6705 |
| 29 | (fl adj2 measure*).ti,ab. | 339 |
| 30 | ((fetal or foetal or fetus* or foetus) adj2 biometr*).ti,ab. | 1371 |
| 31 | 12 or 13 or 14 or 15 or 16 or 17 or 18 or 19 or 20 or 21 or 22 or 23 or 24 or 25 or 26 or 27 or 28 or 29 or 30 | 66071 |
| 32 | 5 and 11 and 31 | 4149 |
| 33 | third trimester pregnancy/ or second trimester pregnancy/ | 47702 |
| 34 | ((second or 2nd or middle) adj3 trimester?).ti,ab. | 28746 |
| 35 | ((third or 3rd or late or last or final) adj3 trimester?).ti,ab. | 32874 |
| 36 | ((late or 2nd half or second half) adj2 pregnanc*).ti,ab. | 13419 |
| 37 | ((14* or 15* or 16* or 17* or 18* or 19* or 20* or 21* or 22* or 23* or 24* or 25* or 26* or 27* or 28* or 29* or 30* or 31* or 32* or 33* or 34* or 35* or 36* or 37* or 38* or 39* or 40* or 41* or 42*) adj2 week?).ti,ab. | 413228 |
| 38 | ((fourteen* or fifteen* or sixteen* or seventeen* or eighteen* or nineteen* or twenty or twentieth or thirty or thirtieth or forty or fortieth) adj3 week?).ti,ab. | 14742 |
| 39 | 33 or 34 or 35 or 36 or 37 or 38 | 478243 |
| 40 | 32 and 39 | 2709 |
| 41 | 11 and 31 | 9851 |
| 42 | limit 41 to "reviews (maximizes specificity)" | 142 |
| 43 | 40 or 42 | 2825 |
| 44 | (exp animals/ or nonhuman/) not human/ | 6751962 |
| 45 | 43 not 44 | 2765 |

Cochrane Library (Wiley)[Issue 4 of 12, April 2021]

| ID | Search |
| --- | --- |
| #1 | MeSH descriptor: [Ultrasonography, Prenatal] explode all trees |
| #2 | ((((ultrasound or ultrasonogra* or ultra-sound or ultra-sonogra* or sonogra* or echograph* or echogram?) and (fetal or foetal or fetus* or foetus or prenatal* or pre-natal*)))):ti,ab,kw |
| #3 | #1 or #2 |
| #4 | MeSH descriptor: [Gestational Age] explode all trees |
| #5 | MeSH descriptor: [Fetal Development] this term only |
| #6 | ("gestational age" or sga):ti,ab,kw |
| #7 | (((fetal or foetal or fetus* or foetus or embryo*) NEAR/2 (growth OR size OR development))):ti,ab,kw OR (((fetal or foetal or fetus* or foetus or menstrua*) NEAR/2 (age or date or dating))):ti,ab,kw |
| #8 | #4 or #5 or #6 or #7 |
| #9 | MeSH descriptor: [Pregnancy Trimester, Third] explode all trees |
| #10 | MeSH descriptor: [Pregnancy Trimester, Second] explode all trees |
| #11 | ((((third or 3rd or late or last or final or 2nd or second or middle) NEAR/3 trimester*))):ti,ab,kw OR (((late or "second half" or "2nd half") NEAR/2 pregnanc*)):ti,ab,kw |
| #12 | (((14* or 15* or 16* or 17* or 18* or 19* or 20* or 21* or 22* or 23* or 24* or 25* or 26* or 27* or 28* or 29* or 30* or 31* or 32* or 33* or 34* or 35* or 36* or 37* or 38* or 39* or 40* or 41* or 42*) NEAR/2 week*)):ti,ab,kw |
| #13 | (((fourteen* or fifteen* or sixteen* or seventeen* or eighteen* or nineteen* or twenty or twentieth or thirty or thirtieth or forty or fortieth) NEAR/3 week*)):ti,ab,kw |
| #14 | #9 or #10 or #11 or #12 or #13 |
| #15 | (((cerebel* or transcerebel*) and diameter*)):ti,ab,kw OR ((head NEAr/2 (circumference or biometr*))):ti,ab,kw OR (cephalometry):ti,ab,kw OR (((biparietal or bi-parietal) NEAR/2 (diameter* or measure* or biometr*))):ti,ab,kw OR (bpd NEAR/2 measure*):ti,ab,kw |
| #16 | (((fundal or symph*) NEAR/2 height)):ti,ab,kw |
| #17 | (((occipitofrontal or occipito-frontal) NEAR/2 (diameter* or measure* or biometr*))):ti,ab,kw OR ((((biparietal or bi-parietal) NEAR/5 (occipitofrontal or occipito-frontal)) and (diameter* or ratio*))):ti,ab,kw OR (((abdomen or abdominal) NEAR/2 (circumference or measure* or biometr*))):ti,ab,kw OR (((femur* or femoral) NEAR/2 (length* or measure* or biometr*))):ti,ab,kw OR (((ac or fl) NEAR/2 measure*)):ti,ab,kw |
| #18 | (((fetal or foetal or fetus* or foetus) NEAR/2 biometr*)):ti,ab,kw |
| #19 | #15 or #16 or #17 or #18 |
| #20 | #3 and #8 and #14 and #19 |

Science Citation Index & Conference Proceedings Citation Index (Web of Science Core Collection [1900-present]

| # 10 | 1,385 | #9 AND #5 AND #2 AND #1 |
| --- | --- | --- |
| # 9 | 263,069 | #8 OR #7 OR #6 |
| # 8 | 227,278 | TS=((14 or 15 or 16 or 17 or 18 or 19 or 20 or 21 or 22 or 23 or 24 or 25 or 26 or 27 or 28 or 29 or 30 or 31 or 32 or 33 or 34 or 35 or 36 or 37 or 38 or 39 or 40 or 41 or 42) NEAR/2 week*) |
| # 7 | 9,447 | TS=(((fourteen* or fifteen* or sixteen* or seventeen* or eghteen* or nineteen* or twenty or twentieth or thirty or thirtieth or fort* or fortieth) NEAR/3 week*)) |
| # 6 | 37,799 | TS=(((third or 3rd or late or last or final or second or 2nd or middle) NEAR/3 trimester*)) OR TS=("late pregnancy" or (("2nd half" OR "second half") NEAR/2 pregnanc*)) |
| # 5 | 32,597 | #4 OR #3 |
| # 4 | 23,105 | TS=(((abdomen or abdominal) NEAR/2 (circumference or measure* or biometr*))) OR TS=((ac NEAR/2 measure*)) OR TS=(((femur* or femoral) NEAR/2 (length* or measure* or biometr*))) OR TS=((fl NEAR/2 measure*)) OR TS=((fetal or foetal or fetus* or foetus) NEAR/2 biometr*) |
| # 3 | 10,726 | TS=(((cerebel* or transcerebel*) and diameter*)) OR TS=(cephalometry) OR TS=((head NEAR/2 (circumference OR biometr*))) OR TS=(((biparietal or bi-parietal) NEAR/2 (diameter* or measure* or biometr*))) OR TS=((bpd NEAR/2 measure*)) OR TS=(((occipitofrontal or occipito-frontal) NEAR/2 (diameter* or measure* or biometr*))) OR TS=((((biparietal or bi-parietal) NEAR/5 (occipitofrontal or occipito-frontal)) and (diameter* or ratio*))) OR TS= ((fundal or symph*) NEAR/2 height) |
| # 2 | 101,737 | TS=(("gestational age") OR ((fetal or foetal or fetus* or foetus) NEAR/2 (growth or development or size)) OR ((fetal or foetal or fetus* or foetus* or menstrua*) NEAR/2 (age OR date OR dating))) |
| # 1 | 35,050 | TS=(((((ultrasound or ultrasonogra* or ultra-sound or ultra-sonogra* or sonogra* or echograph* or echogram?) and (fetal or foetal or fetus* or foetus or prenatal* or pre-natal*))))) |
