# Supplementary material for: Second and third trimester estimation of gestational age using ultrasound or maternal symphysis‐fundal height measurements: A systematic review
Source: BJOG. 2022 Mar 10;129(9):1447–58. doi: 10.1111/1471-0528.17123 (PMC9545821; doi:10.1111/1471-0528.17123)
Supplement: Supplementary file 3 — Appendix S3 [file BJO-129-1447-s001.docx]

| Author | Year | Country | Country income | Measurement | Number of GA formulae | Number of women | Number of scans | Weeks | Dating method | Design and analysis | Data Collection | Inappropriate exclusion of statistical outliers | Quality score | Quality score (%) | |
| --- | --- | --- | --- | --- | --- | --- | --- | --- | --- | --- | --- | --- | --- | --- | --- |
| Abdalla et al^16^ | 2014 | Sudan | Lower middle | UCD | 2 | 50 | unclear | unknown | LMP | NR | prospective | unclear | 5 | | 17 |
| Agwuna et al^17^ | 2016 | Nigeria | Lower middle | PT | 3 | 627 | 626 | 14-40 | LMP | CS | prospective | y | 10 | | 34 |
| Akhtar et al^18^ | 2011 | Pakistan | Lower middle | BPDoi, HC, AC, FL | 4 | 600 | 1599 | 13-40 | Mixed | L | retrospective | y | 6 | | 21 |
| Akram et al^19^ | 2019 | Pakistan | Lower middle | LKL, RKL | 2 | 399 | 399 | 20-38 | Other | CS | prospective | unclear | 8 | | 28 |
| Al Hinsawi et al^20^ | 2020 | Iraq | Upper middle | LKL, RKL | 2 | 150 | 150 | 17-40 | Other | CS | prospective | unclear | 6 | | 21 |
| Altman et al^21^ | 1997 | UK | High | BPDoo, BPDoi, HC, TCD, FL | 5 | 663 | 490 | 12-42 | LMP | CS | prospective | n | 19 | | 66 |
| AMANHI^68^ | 2020 | Bangladesh, Pakistan, Tanzania | Low & lower middle | BPDoi, TCD, AC, FL | 4 | 1611 | 2790 | 24-36+6 | CRL | L | prospective | n | 19 | | 66 |
| Azagidi et al^22^ | 2020 | Nigeria | Lower middle | PT | 3 | 400 | 380 | 11-40 | LMP | CS | prospective | y | 7 | | 24 |
| Briceno et al^23^ | 2013 | Colombia | Upper middle | BPDoi, HC, AC, FL | 4 | 790 | 790 | 12-40 | LMP confirmed by CRL | CS | prospective | y | 19 | | 66 |
| Chang CH et al^24^ | 2000 | Taiwan | High | TCD, CAPD | 3 | 223 | 223 | 20-40 | LMP | CS | unclear | unclear | 9 | | 31 |
| Chang CH et al^25^ | 1998 | Taiwan | High | HC | 1 | 2025 | 2025 | 16-41 | LMP | CS | unclear | y | 10 | | 34 |
| Chang CH et al^26^ | 1996 | Taiwan | High | BPDoi | 1 | 2077 | 2077 | 16-41 | LMP | CS | unclear | y | 10 | | 34 |
| Chang CH et al^27^ | 1997 | Taiwan | High | OFDoo | 1 | 2048 | 2048 | 16-41 | LMP | CS | unclear | y | 10 | | 34 |
| Chang FM et al^28^ | 1997 | Taiwan | High | TL | 1 | 2215 | 2215 | 17-40 | LMP | CS | unclear | y | 12 | | 41 |
| Chang FM et al^29^ | 1997 | Taiwan | High | FbL | 1 | 2184 | 2184 | 17-40 | LMP | CS | unclear | y | 12 | | 41 |
| Chavez et al^30^ | 2004 | USA | High | TCD | 1 | 24026 | 24114 | 14-38 | LMP | CS | retrospective | unclear | 14 | | 48 |
| Chervenak et al^31^ | 1998 | USA | High | BPDoi, HC, AC, FL | 10 | 152 | 152 | 14-22 | IVF | CS | retrospective | y | 12 | | 41 |
| Daloee et al^33^ | 2020 | Iran | Upper middle | IlL, IsL | 3 | 1179 | 1178 | 9-41 | LMP | CS | prospective | unclear | 9 | | 31 |
| Dare et al^32^ | 2004 | UK | High | BPDoo, FL | 2 | 716 | 716 | unknown | LMP confirmed by CRL | CS | prospective | unclear | 7 | | 24 |
| De Crespigney et al^34^ | 1989 | Australia | High | BPDoi | 1 | 677 | 677 | 12+4-40 | LMP | CS | prospective | y | 11 | | 38 |
| Dilmen et al^35^ | 1995 | Turkey | Upper middle | BPD, AC, FL, ScL | 4 | 343 | 343 | 16-41 | LMP | CS | prospective | y | 8 | | 28 |
| Edevbie et al^36^ | 2018 | Nigeria | Lower middle | MKL | 3 | 400 | 400 | 20-41 | Mixed | CS | prospective | y | 8 | | 28 |
| Eze et al^38^ | 2017 | Nigeria | Lower middle | TCD | 3 | 697 | 697 | 14-40 | LMP | CS | prospective | unclear | 10 | | 34 |
| Eze et al^37^ | 2020 | Nigeria | Lower middle | TCD | 3 | 257 | 257 | 16-40 | LMP confirmed by CRL | CS | prospective | unclear | 8 | | 28 |
| Gameraddin et al^39^ | 2014 | Sudan | Lower middle | FtL | 1 | 100 | 100 | T2&T3 | LMP | CS | prospective | y | 6 | | 21 |
| Ghai et al^40^ | 2011 | India | Lower middle | BPDoi | 1 | 25 | 58 | 12-40 | LMP | mixed | unclear | unclear | 4 | | 14 |
| Gilani et al^41^ | 2018 | Pakistan | Lower middle | UCD | 2 | 384 | 384 | unknown | Other | CS | prospective | unclear | 5 | | 17 |
| Gupta et al^42^ | 2013 | India | Lower middle | BPD, HC, AC, FL, MKL | 7 | 715 | unclear | 27-38 | unknown | CS | unclear | y | 4 | | 14 |
| Hadlock et al^43^ | 1982 | USA | High | HC | 1 | 400 | 400 | 15-41 | LMP | CS | unclear | unclear | 7 | | 24 |
| Hadlock et al^44^ | 1982 | USA | High | BPDoi | 1 | 533 | 533 | 12-40 | LMP | CS | unclear | y | 9 | | 31 |
| Hadlock et al^45^ | 1982 | USA | High | AC | 1 | 400 | 400 | 15-41 | LMP | CS | unclear | unclear | 8 | | 28 |
| Hadlock et al^46^ | 1982 | USA | High | FL | 1 | 338 | 338 | 12-40 | Mixed | CS | unclear | unclear | 9 | | 31 |
| Hadlock et al^47^ | 1983 | USA | High | BPDoi, HC, AC, FL | 15 | 177 | 177 | 28-42 | LMP | CS | unclear | unclear | 6 | | 21 |
| Hadlock et al^48^ | 1984 | USA | High | BPDoi, HC, AC, FL | 15 | 361 | 361 | 14-42 | LMP | CS | unclear | unclear | 8 | | 28 |
| Hanoon et al^49^ | 2020 | Iraq | Upper middle | Abdominal aorta | 1 | 59 | 59 | 34-40 | LMP | CS | prospective | unclear | 8 | | 28 |
| Hebbar et al^50^ | 2018 | India | Lower middle | BPDcc | 1 | 100 | unclear | 12-40 | LMP confirmed by CRL | L | prospective | y | 6 | | 21 |
| Hegab et al^51^ | 2018 | Egypt | Lower middle | BPDoi, HC, AC, FL | 1 | 656 | 656 | 12-41 | LMP confirmed by CRL | CS | unclear | unclear | 8 | | 28 |
| Hemraj et al^52^ | 2016 | India | Lower middle | MKL | 1 | 300 | 300 | 18-39 | Mixed | CS | prospective | y | 7 | | 24 |
| Hemraj et al^53^ | 2017 | India | Lower middle | FtL | 1 | 300 | 300 | 18-39 | Mixed | CS | prospective | y | 6 | | 21 |
| Hill et al^54^ | 1990 | USA | High | TCD | 1 | 675 | 669 | 14-42 | LMP | CS | unclear | unclear | 7 | | 24 |
| Hill et al^111^ | 1992 | USA | High | BPDoi, HC, AC, FL | 15 | 265 | unclear | 13-43 | unknown | CS | unclear | y | 3 | | 10 |
| Holanda-Filho et al^55^ | 2011 | Brazil | Upper middle | TCD | 3 | 184 | 184 | 13-40 | unknown | CS | prospective | unclear | 8 | | 28 |
| Honarvar et al^56^ | 2000 | Iran | Upper middle | FL | 1 | 900 | 900 | 14-40 | LMP confirmed by CRL | mixed | prospective | unclear | 5 | | 17 |
| Jiang et al^57^ | 2013 | China | Upper middle | BPDoi, AC, FL | 3 | 6832 | 6832 | 16-41 | LMP confirmed by CRL | CS | prospective | y | 8 | | 28 |
| Johnsen et al^58^ | 2004 | Norway | High | BPDoo, HC | 2 | 642 | 642 | 10-24 | LMP | CS | prospective | unclear | 18 | | 62 |
| Johnsen et al^59^ | 2005 | Norway | High | FL | 1 | 636 | 636 | 10-26 | LMP | CS | prospective | unclear | 19 | | 66 |
| Kapoor et al^60^ | 2016 | India | Lower middle | PT | 1 | 310 | 310 | 11-40 | mixed | CS | prospective | unclear | 7 | | 24 |
| Karabulut et al^61^ | 2001 | Turkey | Upper middle | BPD, HC, AC, FL, SaL | 7 | 186 | 186 | 14-40 | LMP | CS | prospective | y | 8 | | 28 |
| Kaul et al^62^ | 2011 | India | Lower middle | BPDoi, HC, AC, FL, MKL | 7 | 98 | 490 | 24-38 | LMP confirmed by CRL | L | prospective | y | 9 | | 31 |
| Konje et al^63^ | 2002 | UK | High | BPDoi, HC, AC, FL, MKL | 11 | 73 | 584 | 24-38 | LMP confirmed by CRL | L | prospective | unclear | 12 | | 41 |
| Lee et al^2^ | 2020 | Bangladesh | Lower middle | SFH | 1 | 1486 | 3336 | 24-42 | ultrasound <20/40 | L | prospective | unclear | 12 | | 43 |
| Leung et al^64^ | 2008 | Hong Kong | High | BPDoi, BPDoo, HC, FL | 4 | 709 | 709 | 12-40 | LMP confirmed by CRL | CS | prospective | unclear | 18 | | 62 |
| Mahale et al^65^ | 2019 | India | Lower middle | BPD, HC, AC, FL, MKL | 6 | 100 | unclear | 28-41 | LMP | CS | prospective | unclear | 3 | | 10 |
| Mahmoud et al^66^ | 2013 | Sudan | Lower middle | TCD | 1 | 50 | 50 | 15-37 | Mixed | CS | prospective | unclear | 7 | | 24 |
| Marinho et al^67^ | 1987 | Nigeria | Lower middle | FL | 1 | 947 | 1097 | 14-40 | LMP | mixed | unclear | unclear | 6 | | 21 |
| Mercer et al^69^ | 1987 | Canada | High | FtL | 1 | unclear | unclear | 11-43 | LMP | NR | unclear | unclear | 7 | | 24 |
| Mongelli et al^70^ | 2005 | Singapore | High | HC, FL | 3 | unclear | unclear | 26-41 | IVF | CS | retospective | y | 9 | | 31 |
| Nwadike et al^71^ | 2015 | Nigeria | Lower middle | BND | 1 | 320 | 320 | 13-40 | unknown | CS | prospective | y | 7 | | 24 |
| Ohagwu et al^72^ | 2009 | Nigeria | Lower middle | PT | 3 | 730 | 730 | 7-41 | Mixed | CS | prospective | y | 8 | | 28 |
| Orji et al^73^ | 2014 | Nigeria | Lower middle | TCD | 1 | 450 | 450 | 13-42 | LMP | CS | prospective | unclear | 10 | | 34 |
| Osho et al^74^ | 2019 | Nigeria | Lower middle | BPDoi, HC, AC, FL, MKL, MKTD, MKAPD | 12 | 470 | 470 | 28-42 | Mixed | CS | prospective | y | 7 | | 24 |
| Osinusi et al^75^ | 1989 | Nigeria | Lower middle | AC | 1 | 242 | 285 | 16-40 | LMP | mixed | prospective | unclear | 4 | | 14 |
| Osinusi et al^76^ | 1990 | Nigeria | Lower middle | HC | 1 | 242 | 285 | 16-40 | LMP | mixed | prospective | unclear | 3 | | 10 |
| Ott et al^77^ | 1985 | USA | High | BPDoi, HC, AC, FL | 4 | 210 | 210 | 18-42 | LMP | CS | unclear | y | 6 | | 21 |
| Ott et al^78^ | 1994 | USA | High | BPDoi, HC, AC, FL | 2 | 320 | 320 | 15-42 | CRL | CS | retrospective | unclear | 5 | | 17 |
| Ozat et al^79^ | 2011 | Turkey | Upper middle | BPDoi, HC, AC, FL, SaL | 5 | 2184 | unclear | 16-40 | LMP confirmed by CRL | CS | prospective | y | 10 | | 34 |
| Papageorghiou et al^80^ | 2016 | Brazil, China, India, Italy, Kenya, Oman, UK, USA | Mixed | HC, FL | 2 | 4229 | 4229 | 14-42 | LMP confirmed by CRL | CS | prospective | n | 28 | | 97 |
| Papageorghiou et al^81^ | 2016 | Brazil, China, India, Italy, Kenya, Oman, UK, USA | Mixed | SFH | 1 | 4239 | 20023 | 14-42 | LMP confirmed by CRL | L | prospective | n | 25 | | 89 |
| Persson et al^82^ | 1986 | Sweden | High | BPD, OFD, AD, FL | 7 | 14 | 67 | 11-26 | LMP confirmed by CRL | L | prospective | unclear | 11 | | 38 |
| Persson et al^83^ | 1986 | Sweden | High | BPDoi, OFDoi, AD, FL | 4 | 19 | 167 | 11-40 | LMP confirmed by CRL | L | prospective | unclear | 9 | | 31 |
| Piantelli et al^84^ | 1994 | Italy | High | BPDoi, TCD, FL, HL, BND | 5 | 72 | >288 | 7-40 | LMP | L | prospective | y | 9 | | 31 |
| Quinlan et al^85^ | 1982 | USA | high | FL | 1 | 125 | 130 | 14-36 | LMP | mixed | unclear | unclear | 9 | | 31 |
| Reddy et al^86^ | 2017 | India | Lower middle | TCD | 1 | 100 | 100 | 15-40 | LMP | CS | prospective | y | 6 | | 21 |
| Rodriguez-Sibaja et al^87^ | 2020 | Brazil, India, Italy, Kenya, UK | Mixed | TCD | 1 | 1130 | 3016 | 14-42 | LMP confirmed by CRL | L | prospective | n | 23 | | 79 |
| Sabbagha et al^88^ | 1974 | USA | High | BPDoo | 1 | unclear | 744 | 20-40 | LMP | NR | unclear | y | 4 | | 14 |
| Satish Prasad et al^89^ | 2014 | India | Lower middle | BPDcc, HC, TCD, FL | 4 | 80 | 80 | 15-40 | unclear | CS | unclear | unclear | 6 | | 21 |
| Schluter et al^90^ | 2007 | Australia | High | BPDoi, HC, AC, FL | 4 | 17660 | 20555 | 15-41 | LMP | CS | prospective | n | 14 | | 48 |
| Schmidt et al^91^ | 1985 | Germany | High | FL | 2 | 595 | 595 | 12-42 | LMP | CS | prospective | y | 12 | | 41 |
| Selbing et al^92^ | 1985 | Sweden | High | BPDoi | 1 | 970 | 970 | 9-22 | CRL | CS | unclear | unclear | 12 | | 41 |
| Senapati et al^93^ | 2016 | India | Lower middle | BPD, HC, AC, FL | 4 | 170 | 170 | 13-40 | LMP | CS | prospective | y | 2 | | 7 |
| Sherer et al^94^ | 1993 | USA | High | SaL | 1 | 506 | 506 | 15-41 | LMP | CS | prospective | y | 10 | | 34 |
| Skupski et al^95^ | 2017 | USA | High | BPDoi, HC, AC, FL | 1 | 2802 | 2802 | 14-40 | LMP confirmed by CRL | CS | prospective | unclear | 19 | | 66 |
| Sun et al^96^ | 2020 | China | Upper middle | BPDoi, HC, AC, FL | 1 | 2908 | 2908 | 15-40 | LMP confirmed by CRL | CS | prospective | unclear | 16 | | 55 |
| Swapnil et al^97^ | 2021 | India | Lower middle | BPDoi, HC, AC, FL | 1 | 150 | 150 | 13-28 | LMP | CS | unclear | unclear | 4 | | 14 |
| Tongsong et al^98^ | 1992 | Thailand | Upper middle | BND | 1 | 555 | 555 | 14-40 | LMP | CS | prospective | y | 9 | | 31 |
| Tse et al^99^ | 1984 | Hong Kong | High | BPDoi, FL | 2 | 50 | 50 | 28-42 | LMP | CS | unclear | unclear | 11 | | 38 |
| Uerpairojkit et al^100^ | 2001 | Thailand | Upper middle | TCD | 1 | 153 | 699 | 14-40 | LMP | CS | prospective | y | 11 | | 38 |
| Ugur et al^101^ | 2016 | Turkey | Upper middle | MKL | 1 | 180 | unclear | 24-39 | LMP | NR | prospective | y | 3 | | 10 |
| Varol et al^102^ | 2001 | Turkey | Upper middle | BPD, AC, FL | 10 | 1411 | 1411 | 13-40 | LMP | CS | retrospective | y | 6 | | 21 |
| Varsha et al^103^ | 2020 | India | Lower middle | HC | 1 | 132 | 132 | 29-42 | LMP | CS | unclear | unclear | 3 | | 10 |
| Verburg et al^104^ | 2008 | Netherlands | High | BPDoo | 1 | 3760 | 10470 | 12-30 | LMP | L | prospective | y | 17 | | 59 |
| Viveki et al^105^ | 2019 | India | Lower middle | AC | 1 | 768 | 768 | 20-38 | LMP | CS | retrospective | y | 8 | | 28 |
| Warda et al^106^ | 1985 | USA | High | FL | 1 | 254 | 254 | 13-39 | LMP | CS | unclear | y | 12 | | 41 |
| Westerway et al^107^ | 2000 | Australia | High | BPDoi, OFDoi, AC, FL | 2 | 3800 | 3800 | 11-40 | LMP | CS | unclear | unclear | 6 | | 21 |
| White et al^108^ | 2012 | Thailand | Upper middle | SFH | 3 | 2437 | 7476 | unknown | Mixed | L | prospective | unclear | 7 | | 25 |
| Yagel et al^109^ | 1986 | Israel | High | BPD, FL | 1 | 1087 | 1123 | 14-41 | LMP | mixed | unclear | y | 3 | | 10 |
| Yarkoni et al^110^ | 1985 | USA | High | CL | 1 | 85 | 85 | 15-40 | unknown | CS | prospective | unclear | 12 | | 41 |
| BPD, Biparietal diameter; oo, outer to outer; oi, outer to inner; cc, centre of near brightest echo to centre of far brightest echo; OFD, occipito frontal diameter; HC, head circumference; TCD, transcerebellar diameter; AC, abdominal circumference; FL, femur length; UCD, umbilical cord diameter; PT, placental thickness; RKL, right kidney length; LKL, left kidney length; MKL, mean kidney length; CAPD, cerebellar antero-posterior diameter; TL, tibia length; FbL, fibula length; IlL, iliac length; IsL, ischial length; ScL, scapula length; SaL, sacral length; FtL, foot length; SFH, symphysis fundal height; BND, binoccular distance; MKTD, mean kidney transverse diameter; MKAPD, mean kidney antero posterior diameter; HL, humerus length; CL, clavicular length; CS, cross-sectional; L, longitudinal; NR, not reported or unable to ascertain | | | | | | | | | | | | | | | |
