# Supplementary material for: Second and third trimester estimation of gestational age using ultrasound or maternal symphysis‐fundal height measurements: A systematic review
Source: BJOG. 2022 Mar 10;129(9):1447–58. doi: 10.1111/1471-0528.17123 (PMC9545821; doi:10.1111/1471-0528.17123)
Supplement: Supplementary file 9 — Data S6 [file BJO-129-1447-s004.docx]

| ICMJE DISCLOSURE FORM | |
| --- | --- |
| **Date:** | 12/16/2021 |
| **Your Name:** | Aris Papageorghiou |
| **Manuscript Title:** | **Second and third trimester estimation of gestational age using ultrasound or maternal symphysis-fundal height measurements: A systematic review** |
| **Manuscript Number (if known):** | BJOG-21-1651 |
| In the interest of transparency, we ask you to disclose all relationships/activities/interests listed below that are related to the content of your manuscript. “Related” means any relation with for-profit or not-for-profit third parties whose interests may be affected by the content of the manuscript. Disclosure represents a commitment to transparency and does not necessarily indicate a bias. If you are in doubt about whether to list a relationship/activity/interest, it is preferable that you do so.  The author’s relationships/activities/interests should be defined broadly. For example, if your manuscript pertains to the epidemiology of hypertension, you should declare all relationships with manufacturers of antihypertensive medication, even if that medication is not mentioned in the manuscript.  In item #1 below, report all support for the work reported in this manuscript without time limit. For all other items, the time frame for disclosure is the past 36 months. | |

|  | | | **Name all entities with whom you have this relationship or indicate none (add rows as needed)** | **Specifications/Comments (e.g., if payments were made to you or to your institution)** |
| --- | --- | --- | --- | --- |
| **Time frame: Since the initial planning of the work** | | | | |
| **1** | All support for the present manuscript (e.g., funding, provision of study materials, medical writing, article processing charges, etc.)  **No time limit for this item.** | | \|  \| **None** \| \| --- \| --- \|  \| I am supported by the National Institute for Health Research (NIHR) Oxford Biomedical Research Centre (BRC). \| Payment to my institution \| \| --- \| --- \| \|  \|  \| \|  \| Click the tab key to add additional rows. \| | |
| **Time frame: past 36 months** | | | | |
| **2** | | Grants or contracts from any entity (if not indicated in item #1 above). | \|  \| **None** \| \| --- \| --- \|  \| I hold a number of research grants from the NIHR / HTA, NIHR Oxford Biomedical Research Centre, EPSRC, GCRF, NIH and Bill and Melinda Gates Foundation. \| These grants fund research studies. They pay part of my salary and overheads to my institution. \| \| --- \| --- \| \|  \|  \| \|  \|  \| | |
| **3** | | Royalties or licenses | \|  \| **None** \| \| --- \| --- \|  \| I receive royalties for published books (Oxford University Press) \| Payments made to me \| \| --- \| --- \| \|  \|  \| \|  \|  \| | |
| **4** | | Consulting fees | \|  \| **None** \| \| --- \| --- \|  \| I am a senior advisor for Intelligent Ultrasound. This is undertaken via Oxford University Innovations which manages consulting activities of University staff. \| Payments made to me \| \| --- \| --- \| \|  \|  \| \|  \|  \| \|  \|  \| | |
| **5** | | Payment or honoraria for lectures, presentations, speakers bureaus, manuscript writing or educational events | \|  \| **None** \| \| --- \| --- \|  \| I have received honoraria for lectures in the field of fetal ultrasound from Capital Medical University, Beijing; from GE Healthcare; and Samsung Medison. \| Payments made to me \| \| --- \| --- \| \|  \|  \| \|  \|  \| | |
| **6** | | Payment for expert testimony | \|  \| **None** \| \| --- \| --- \|  \|  \|  \| \| --- \| --- \| \|  \|  \| \|  \|  \| | |
| **7** | | Support for attending meetings and/or travel | \|  \| **None** \| \| --- \| --- \|  \| I have received travel costs (no honoraria) for attending meetings by the Ministry of Health, Cyprus; Capital Medical University, Beijing; and the East Virginia Medical School, USA. \| Expenses covered but no honorarium \| \| --- \| --- \| \|  \|  \| \|  \|  \| | |
| **8** | | Patents planned, issued or pending | \|  \| **None** \| \| --- \| --- \|  \| I hold a patent entitled “A system and method are provided to automatically categorize biological and medical images” US10762630B2 \| No \| \| --- \| --- \| \|  \|  \| \|  \|  \| | |
| **9** | | Participation on a Data Safety Monitoring Board or Advisory Board | \|  \| **None** \| \| --- \| --- \|  \|  \|  \| \| --- \| --- \| \|  \|  \| \|  \|  \| | |
| **10** | | Leadership or fiduciary role in other board, society, committee or advocacy group, paid or unpaid | \|  \| **None** \| \| --- \| --- \|  \| I am Deputy Editor in Chief for BJOG for which I receive a stipend. \| Payment made to me \| \| --- \| --- \| \|  \|  \| \|  \|  \| | |
| **11** | | Stock or stock options | \|  \| **None** \| \| --- \| --- \|  \| I am a co-founder of and hold stock in Intelligent Ultrasound, a University spin–out company. \|  \| \| --- \| --- \| \|  \|  \| \|  \|  \| | |
| **12** | | Receipt of equipment, materials, drugs, medical writing, gifts or other services | \|  \| **None** \| \| --- \| --- \|  \|  \|  \| \| --- \| --- \| \|  \|  \| \|  \|  \| | |
| **13** | | Other financial or non-financial interests | \|  \| **None** \| \| --- \| --- \|  \| Part of my time I work in private medical practice. \|  \| \| --- \| --- \| \|  \|  \| \|  \|  \| | |
|  | |  |  | |
| **Please place an “X” next to the following statement to indicate your agreement:** | | | | |
|  | | I certify that I have answered every question and have not altered the wording of any of the questions on this form. | | |
